# Imaging in medically refractory epilepsy at 3 Tesla: a 13-year tertiary adult epilepsy center experience

**DOI:** 10.1186/s13244-022-01236-1

**Published:** 2022-06-04

**Authors:** Nicolin Hainc, Mary Pat McAndrews, Taufik Valiante, Danielle M. Andrade, Richard Wennberg, Timo Krings

**Affiliations:** 1 Division of Neuroradiology, Joint Department of Medical Imaging, Toronto Western Hospital, University Health Network, University of Toronto, Toronto, ON Canada; 2 Department of Neuroradiology, Clinical Neuroscience Center, University Hospital Zurich, University of Zurich, Zurich, Switzerland; 3 Krembil Brain Institute, Toronto Western Hospital, University Health Network, University of Toronto, Toronto, ON Canada; 4 Division of Neurosurgery, Department of Surgery, University of Toronto, Toronto, ON Canada; 5 Division of Neurology, Department of Medicine, University of Toronto, Toronto, ON Canada

**Keywords:** Epilepsy, Drug resistant epilepsy, Magnetic resonance imaging

## Abstract

**Objectives:**

MRI negative epilepsy has evolved through increased usage of 3 T and insights from surgically correlated studies. The goal of this study is to describe dedicated 3 T epilepsy MRI findings in medically refractory epilepsy (MRE) patients at a tertiary epilepsy center to familiarize radiologists with an updated spectrum and frequency of potential imaging findings in the adult MRE population.

**Methods:**

Included were all patients with MRE admitted to the epilepsy monitoring unit who were discussed at weekly interdisciplinary imaging conferences at Toronto Western Hospital with MRI studies (3 T with dedicated epilepsy protocol) performed between January 2008 and January 2021. Lesion characterization was performed by two readers based on most likely imaging diagnosis in consensus. Lobes involved per case were recorded.

**Results:**

A total of 738 patients (386 female; mean age 35 years, range 15–77) were included. A total of 262 patients (35.5%) were MRI negative. The most common imaging finding was mesial temporal sclerosis, seen in 132 patients (17.9%), followed by encephalomalacia and gliosis, either posttraumatic, postoperative, postischemic, or postinfectious in nature, in 79 patients (10.7%). The most common lobar involvement (either partially or uniquely) was temporal (341 cases, 58.6%). MRE patients not candidates for surgical resection were included in the study, as were newly described pathologies from surgically correlated studies revealing findings seen retrospectively on reported MRI negative exams (isolated enlargement of the amygdala, temporal pole white matter abnormality, temporal encephalocele).

**Conclusion:**

This study provides an updated description of the spectrum of 3 T MRI findings in adult MRE patients from a tertiary epilepsy center.

## Key points

- Through increased usage of 3 T MRI and insights from surgically correlated MRI negative studies, the definition of MRI negative epilepsy has evolved.
- All patients with medically refractory epilepsy and 3 T MRI studies were included to assess the frequency of imaging findings encountered.
- This study provides a comprehensive description of 3 T MRI findings in adult medically refractory epilepsy patients from a tertiary epilepsy center.

## Introduction

Epilepsy affects 70 million people worldwide, with a median incidence of 50 new cases per 100,000 persons per year [1]. Of these, an estimated 22.5% [2] to 37% [3] go on to develop medically refractory epilepsy (MRE), meaning they have failed adequate trials of at least two antiepileptic drugs to attain seizure freedom. Neurosurgery is potentially curative in selected MRE patients; those with epileptogenic lesions demonstrated on MRI are more likely to become candidates for surgical resection and have better outcomes as compared to “MRI negative” MRE patients [4]. On MRI, epileptogenic lesions are often subtle, with sensitivity decreased through usage of lower field (1.5 T) MRI [5], non-epilepsy tailored MRI protocols, and assessment by non-expert readers [6, 7], yet only a fraction of MRE patients are referred to specialized, tertiary care epilepsy centers with epilepsy monitoring units and dedicated multidisciplinary evaluation for further assessment [8].

The rate of MRI negative epilepsy cases reported by prior studies varies greatly, depending on clinical scenario and type of epilepsy. Prior MRE studies report MRI negative rates ranging from 17 to 43% [9, 10]; notably, these studies were performed using 1.5 T MRI systems and one excluded all non-surgical MRE patients for purposes of histopathological correlation [10]. With respect to other epilepsy patient cohorts, a prospective study of patients presenting with new-onset seizures reported a lesion detection rate of 47%, also using 1.5 T MRI [11]. More recently, a meta-analysis of epilepsy MRI studies based on field strengths ranging from 1.5 T to 7 T found an 83% overall detection rate for temporal lobe epilepsy patients [7]. The goal of our study was to describe dedicated 3 T epilepsy protocol MRI findings in all MRE patients at an adult tertiary epilepsy center, regardless if neurosurgery was performed, to familiarize radiologists with the spectrum and frequency of potential imaging findings in the adult MRE population.

## Materials and methods

### Study cohort

This study was exempt from Research Ethics Board (REB) review as determined by the University Health Network (UHN) Quality Improvement Review Committee (QIRC) (QI ID 21–0211). The study cohort comprised consecutive patients with MRE admitted to the epilepsy monitoring unit who were discussed at weekly interdisciplinary imaging conferences at Toronto Western Hospital with MRI studies between January 2008 and January 2021. Excluded from the study were patients without MRI studies available for review, patients scanned at 1.5 T, patients scanned at 3 T without a dedicated epilepsy MRI protocol, and patients with quality degraded MRI studies.

### MR imaging

Our dedicated epilepsy MRI protocol incorporates 3D sequences with millimetric, isotropic resolutions and 2D T2-weighted, coronal oblique sequences with submillimetric in-plane resolutions, in keeping with recommendations put forth by the International League Against Epilepsy [12]. All studies were performed with our dedicated protocol on 3 T scanners, either a Signa HDxt (GE Healthcare, Chicago, Illinois) or a Skyra (Siemens Healthineers AG, Erlangen, Germany). Imaging parameters were as follows: For GE: axial FLAIR TR/TE 9102.00/141.17 ms, FOV 22.0 × 22.0 cm, matrix 384 × 224, slice thickness 4.0 mm; coronal oblique T2 perpendicular to the hippocampus TR/TE 6500/40 ms, FOV 22.0 × 22.0 cm, matrix 512 × 512, slice thickness 3.0 mm; coronal oblique FLAIR perpendicular to the hippocampus TR/TE 8802/143.34 ms, FOV 22.0 × 22.0 cm, matrix 252 × 224, slice thickness 4.0 mm; sagittal 3D T1 TR/TE 7.13/2.94 ms, FOV 22.0 × 22.0, matrix 256 × 256, slice thickness 1.0 mm; DWI TR/TE 8000.00/81.90 ms, FOV 22.0 × 22.0 cm, matrix 256 × 256, slice thickness 4.0 mm, GRE TR/TE 2000/25 ms, FOV 22.0 × 22.0 cm, matrix 264 × 256, slice thickness 4.0 mm. For Siemens: axial and coronal oblique FLAIR perpendicular to the hippocampus TR/TE 9000/94 ms, FOV 19.9 × 22.0 cm, matrix 256 × 232, slice thickness 4.0 mm; coronal oblique T2 perpendicular to the hippocampus TR/TE 6900/28 ms, FOV 22.0 × 18.6 cm, matrix 432 × 512, slice thickness 3.0 mm; sagittal 3D T1 TR/TE 2300/2.27 ms, FOV 25.0 × 25.0, slice thickness 1.0 mm, matrix 256 × 256; DWI TR/TE 6900/94 ms, FOV 22.0 × 22.0 cm, matrix 160 × 160, slice thickness 4.0 mm, SWI TR/TE 28/20 ms, FOV 18.0 × 23.0 cm, matrix 320 × 230, slice thickness 2.6 mm.

### Imaging findings

All MRI scans were evaluated by two neuroradiologists (T.K. and N.H.) in consensus, with 21 and 8 years of experience, respectively. Lesion characterization was based on most likely imaging diagnosis. Lobes involved per case were listed, and concomitant mesial temporal sclerosis (MTS) was assessed for in all cases.

## Results

### Study cohort

**Fig. 1:**
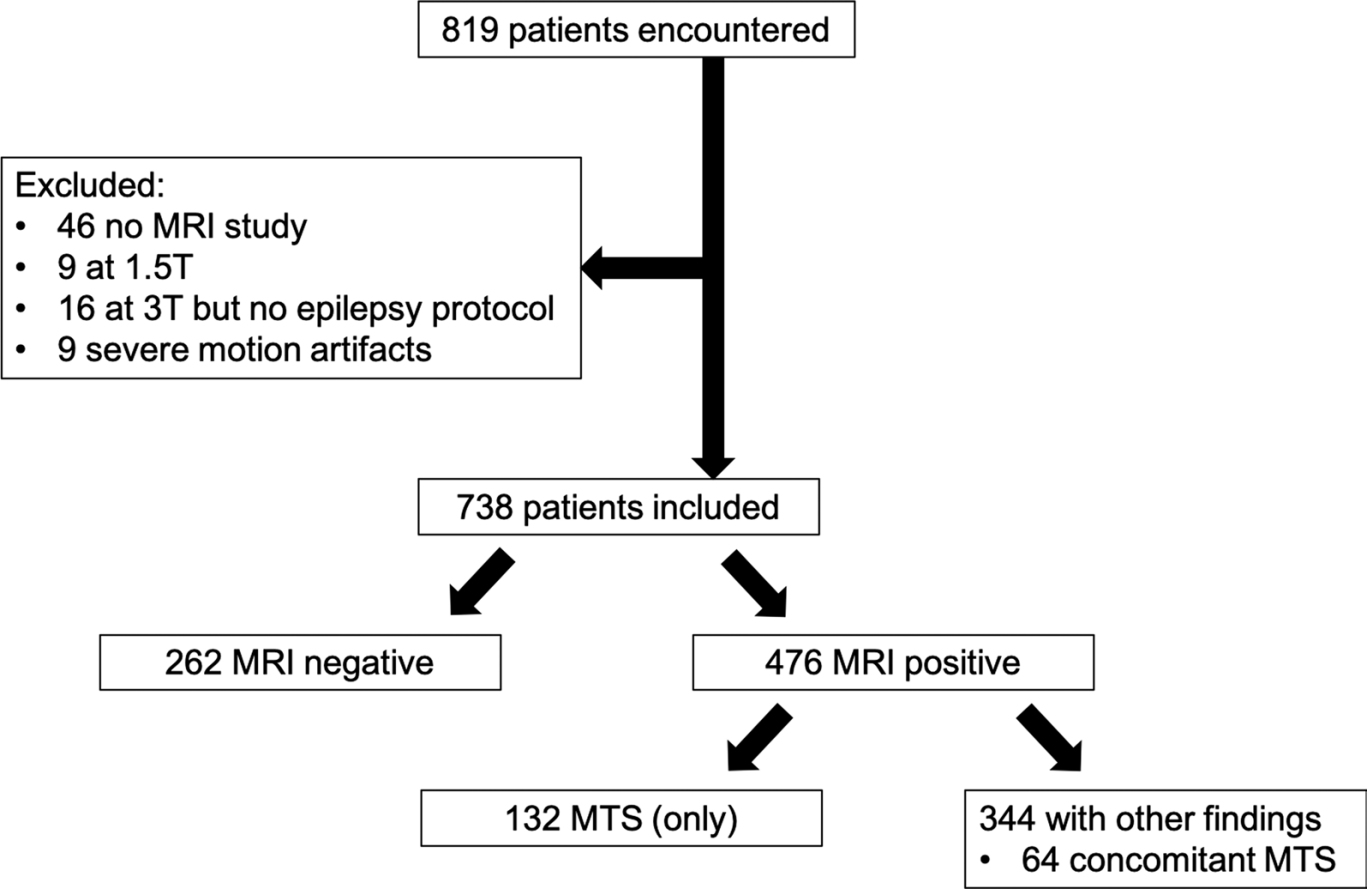
Patients included in study including number of mesial temporal sclerosis (MTS) diagnoses

A total of 819 patients were identified. Of these, 46 had no MRI available for review. A further 9 patients were scanned at 1.5 T, 16 had no dedicated epilepsy protocol at 3 T, and 9 had severe motion artifacts precluding assessment. Thus, 738 patients (386 female, mean age 35 years, range 15–77) with MRI studies ranging from January 2008 to January 2021 were included (Fig. 1).

### Imaging findings

A total of 262 patients (35.5%) had no abnormalities determined on MRI (MRI negative). The most common imaging finding was MTS, seen in 132 patients (17.9%), 20 of which contained bilateral cases of MTS. In the 112 cases of unilateral MTS, the combination of volume loss, T2/FLAIR hyperintensity, and internal architectural distortion was seen in 98 cases (87.5%), followed by volume loss and T2/FLAIR hyperintensity without internal architectural distortion (8 cases, 7.1%), isolated volume loss relative to the opposite side (4 cases, 3.6%), and isolated T2/FLAIR hyperintensity (2 cases, 1.8%). A further 64 patients harbored “dual pathology,” i.e., a separate imaging finding combined with MTS (8.7%). In 53 cases (82.8%), the affected hippocampus was ipsilateral to the concomitant finding, with 5 contralateral MTS cases (7.8%) and 6 bilateral MTS cases (9.4%) in addition to the concomitant finding. The full list of imaging diagnoses can be found in Table 1.

**Table 1:** Complete list of all imaging diagnoses encountered in the cohort

Imaging diagnosis	Number of patients	% of cohort
MRI negative	262	35.50
Mesiotemporal sclerosis	132 (20 bilateral cases)	17.89
Concomitant mesiotemporal sclerosis (dual pathology)	64	8.67
Encephalomalacia and gliosis	79	10.70
Focal cortical dysplasia	47	6.37
Isolated enlargement of the amygdala	40	5.42
Enlarged amygdala with involvement of surrounding structures	8	1.08
Tumor (18 DNET, 11 LGG, 3 Ganglioglioma, 2 PXA, 1 choroid plexus papilloma within the choroid fissure)	35	4.74
Cavernoma	22	2.98
Polymicrogyria	14	1.90
Periventricular nodular heterotopic gray matter	13	1.76
Subcortical nodular heterotopic gray matter	2	0.27
Band heterotopia	6	0.81
Ulegyria	12	1.63
Perinatal hypoxic gliosis/encephalomalacia without ulegyria	7	0.95
Encephalocele	10	1.36
Hippocampal malrotation	9	1.22
White matter abnormalities in the anterior temporal lobe	8	1.08
Cortical siderosis	3	0.41
Mass effect onto hippocampus	3	0.41
Tuberous sclerosis	4	0.54
Rasmussen's encephalitis	3	0.41
Neurocysticercosis	2	0.27
Closed lip schizencephaly	2	0.27
Pachygyria, Hypothalamic hamartoma, Dandy-Walker variant, Dyke-Davidoff-Masson, Diffuse axonal injury with cortical hemorrhage, Hemimegalencephaly, Limbic encephalitis, Neurofibromatosis type I, Diffuse cortical diffusion restriction, Dysmyelination with anteroposterior gradient, Diffuse cortical thinning, Arteriovenous malformation with postradiation gliosis, Pontine osmotic myelinolysis, Remote anterior callosotomy, Meningeoangiomatosis	1 each	0.14

Encephalomalacia and gliosis, either posttraumatic, postoperative, postischemic, or postinfectious, were the second most common finding, found in 79 patients (10.7%). Third most common was focal cortical dysplasia (FCD), found in 47 patients (6.4%), 10 of which had a transmantle band suggestive of Type IIb FCD. Isolated enlargement of the amygdala was found in 40 patients (5.4%) with an additional 8 demonstrating involvement of adjacent structures (7 hippocampus, 1 uncus). 35 patients (4.7%) had tumors (18 dysembryoplastic neuroepithelial tumor (DNET), 11 low grade glioma (LGG), 3 ganglioglioma, 2 pleomorphic xanthoastrocytoma (PXA), 1 choroid plexus papilloma within the choroid fissure). Cavernomas were found in 22 patients (3%). The most common malformation of cortical development apart from FCD was polymicrogyria (14 patients, 1.9%) followed by periventricular nodular gray matter heterotopia (PVNH, 13 patients, 1.8%), band heterotopia (6 patients, 0.8%), subcortical nodular heterotopia (2 patients, 0.3%), and pachygyria (1 subject, 0.1%).

**Fig. 2:**
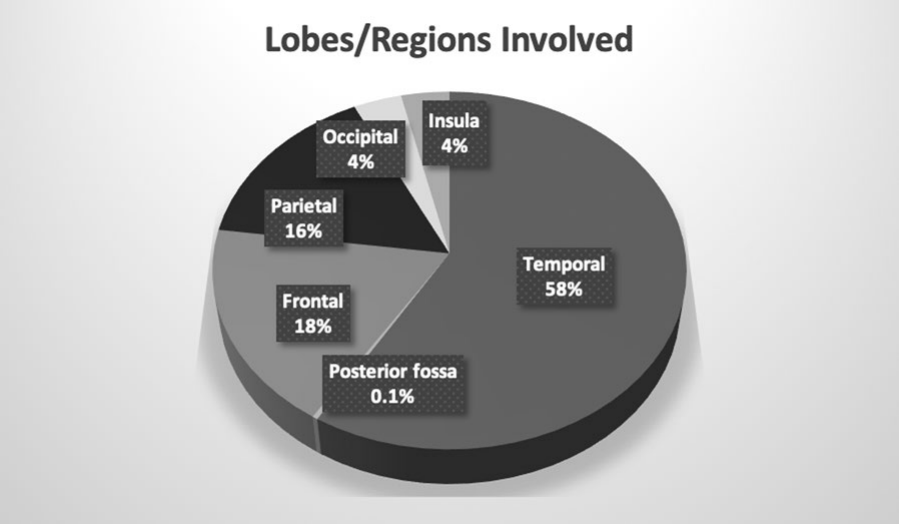
Pie chart of brain lobes/regions involved by the imaging diagnosis

Of the 476 patients with findings on MRI, a total of 582 brain lobes/regions were involved by the pathological entity (Fig. 2). The temporal lobe was involved (partially or uniquely) in 341 cases (58.6%) followed by the frontal lobe (106, 18.2%), parietal lobe (91, 15.6%), occipital lobe (21, 3.6%), and insula (21, 3.6%). The posterior fossa was involved in 2 cases, namely one case of PVNH of the 4^th^ ventricle and one case of osmotic pontine myelinolysis.

## Discussion

In our series of 738 patients with MRE imaged at 3 T, MRI was negative in 35.5% of cases, a finding at the higher end of the previously reported MRE cohort spectrum ranging from 17 to 43% [9, 10]. Differences between our study and other prior epilepsy cohort studies are manifold. First, our study focused on patients with MRE, a cohort that has different imaging-finding frequencies when compared with new-onset seizure cohorts [11, 13]. Second, we included only patients imaged at 3 T, which is known to have higher sensitivities for subtle lesions when compared with 1.5 T [14] through higher resolution, better signal-to-noise ratio, and improved tissue contrast [5, 15, 16]. Finally, all cases of MRE were included, regardless of surgical candidacy, in an attempt to replicate the clinical scenario faced by radiologists in routine practice.

A total of 262 patients (35.5%) had no abnormalities determined on MRI (MRI negative), representing the most common imaging diagnosis in our study. In previous studies, FCD was the most common pathology found among surgically treated MRI negative patients [17–19]. In a large-scale study by Wang et al. spanning 10 years, FCD type I represented the majority of all FCD cases (37/43) in 95 surgical MRI negative patients [17]. FCD type I is known to be illusive on MRI when compared with the more conspicuous FCD type II [20]. While increased cortical thickness, abnormal gyral/sulcal patterns, blurring of the gray-white matter junction and the transmantle sign are all imaging features of FCD type II, they are rarely seen with FCD type I [20]; in some cases, blurring of the gray-white matter junction and cortical thickening has been described in FCD type I [21]. FCD type I is known to be a diffuse, often multilobar pathology [22], and is associated with lobar hypoplasia/atrophy, which may help with identification and localization of these often invisible lesions [20]. Newer pulse sequences [23] and voxel-based morphometry [24] have shown promising results in the detection FCD type I through increased visual conspicuity and quantification. Thus, while FCD was the third most common finding in our study, seen in 47 patients (6.4%), the true number is likely higher based on the studies mentioned above on MRI negative patients.

MTS was the most common pathological finding in our study, seen in 132 patients (17.9%) with an additional 64 patients (9%) harboring dual pathology, i.e., a separate imaging finding and MTS. A study by Urbach et al. reported MTS in 26.2% of their prospective MRI cohort of MRE patients (101/385 patients) while failing to detect MTS in 3 additional cases after histopathological correlation [9]. Notably, entities we included, such as gliosis and encephalomalacia (our second largest entity) were not described in their study, and their cohort included only patients who were considered for surgery, which may explain the discrepancy in the reported frequency of MTS imaging findings. A further study on MRE patients who underwent surgical resection reported 2/64 cases of MTS based on imaging, and further 2/64 cases after histopathology not identified on MRI pre-surgically [10].

**Fig. 3:**
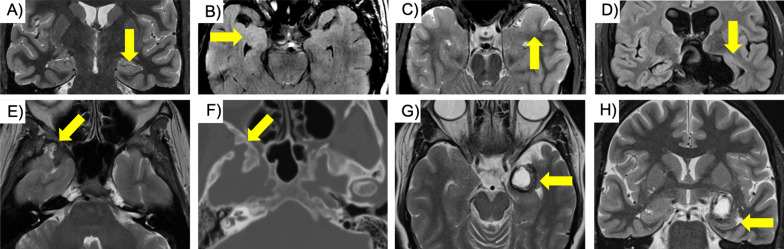
Temporal lobe findings. **a** Subtle sign of left sided MTS with loss of the interdigitations of the pes hippocampi (arrow). **b** Right sided isolated enlargement of the amygdala (arrow). **c** White matter abnormality in the anterior left temporal lobe (arrow). **d** Suprasellar arachnoid cyst with compression of the left hippocampus (arrow). **e** Right temporopolar encephalocele with herniation of brain parenchyma (arrow) through a bony defect in the sphenoid (**f**, arrow, same patient as **e**). **g** Left sided MCA aneurysm with lamellated onion skin appearance (arrow) resulting in inferior displacement and compression of the left hippocampus (**h**, arrow, same patient as **g**)

Dual pathology has been described in the literature to range between 8 and 22% [25], and MTS has been described to have a high prevalence among MRI negative studies, with normal appearing hippocampi reported in up to 29% of proven MTS surgical cases [17, 26]. Clinically, MRI negative patients with MTS had better chances at a seizure-free outcome when compared with MRI negative FCD patients and pathology negative patients [17]**. **MRI findings of MTS range from subtle T2/FLAIR hyperintensity of the hippocampus or flattening of the interdigitations at the superior aspect of the hippocampal head to frank hippocampal volume loss combined with T2/FLAIR hyperintensity [27] and internal architectural distortion [28] (Fig. 3). MTS is commonly due to febrile seizures in childhood and can progress to medically refractory epilepsy in up to 90% of cases, upon which surgical resection is offered [29]. In the case of dual pathology, simultaneous surgical removal of the lesion and the affected hippocampus resulted in best outcome in a study by Li et al. when compared to surgical resection of only one lesion alone [30].

**Fig. 4:**
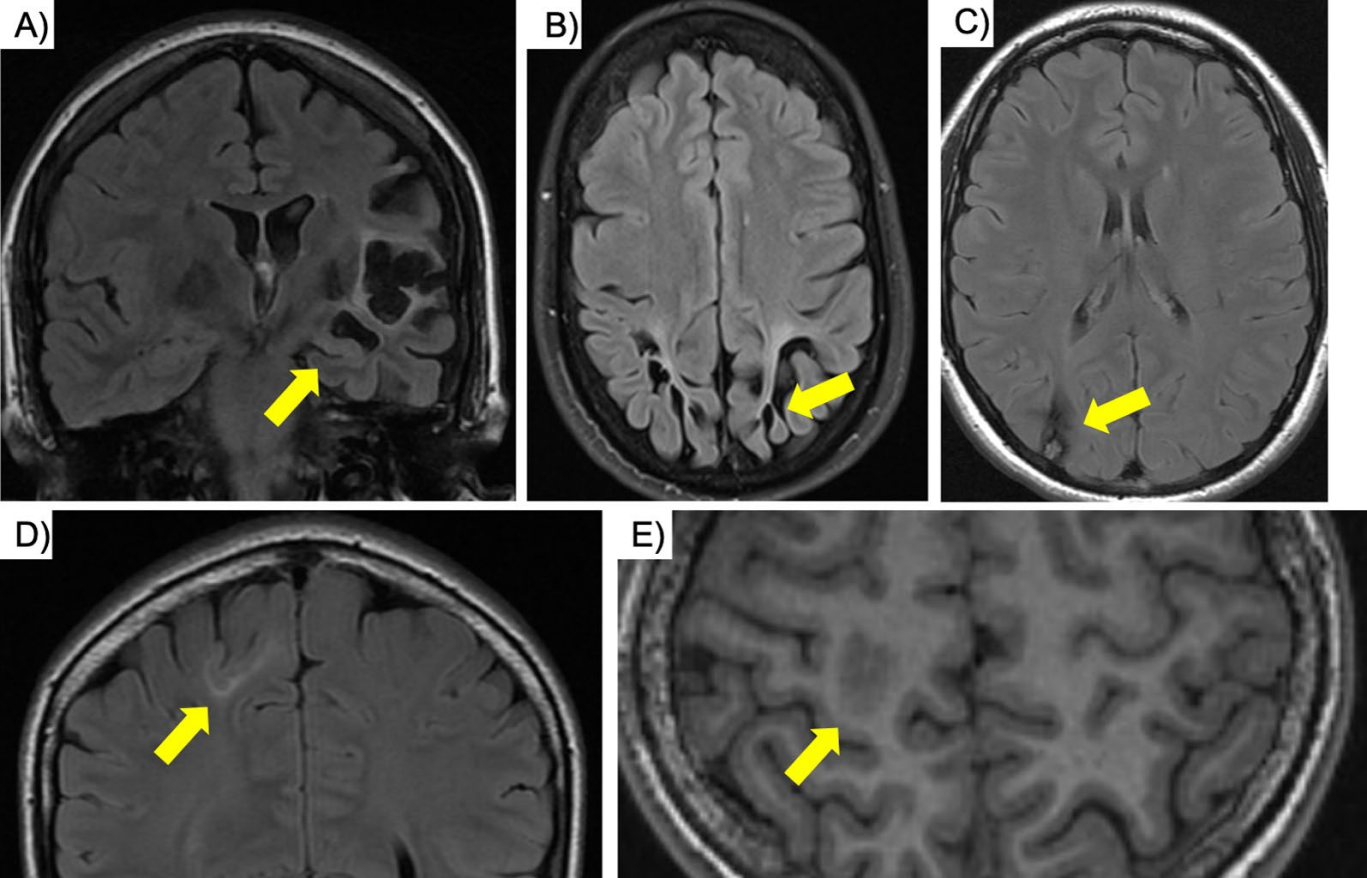
Extratemporal findings: **a** Chronic left MCA infarct with concomitant left MTS (arrow, dual pathology). **b** Adult patient with birth-related findings of biparietal thinning of the white and gray matter with associated gliosis and ulegyria (arrow). **c** Cortically based, right parietal cavernoma with associated hemosiderin staining of the white matter. **d** and **e** (same patient) Focal cortical dysplasia with cortical thickening, blurring and FLAIR Hyperintensity of the gray-white matter junction (arrows)

Encephalomalacia and gliosis comprised the second largest entity in our cohort (79 patients, 10.7%), with etiologies including cerebrovascular disease, trauma, infection, inflammatory disease, and prior surgical resection (Fig. 4). A study by Li et al. reported 8.3% encephalomalacia and gliosis due to infarct and contusion in their cohort of 341 patients using a 1.5 T MRI system [31] and postischemic encephalomalacia and gliosis has been described to be the most common cause of seizures in the elderly [32]. A common pathophysiology amongst the above listed etiologies appears to be cortical gliosis, neuronal loss, changes in neuronal connectivity, and deposition of hemosiderin within the cortex [32, 33]. A subgroup (19 patients, 2.6%) of cerebrovascular disease patients had patterns suggestive of gestational or perinatal ischemia, i.e., periventricular leukomalacia for early gestational insults and biparietal thinning of the white and gray matter with associated gliosis in term neonates. In this subgroup, 12 patients demonstrated ulegyria, or mushroom shaped gyri, formed due to the increased susceptibility to hypoxemia at the depth of the sulci in term neonates. Ulegyria is highly epileptogenic, likely through hyperexcitability of the damaged cortex and through network rearrangements in the surrounding cortex [34], and can be treated surgically [35].

Recently, awareness has increased for other potentially resectable epileptogenic lesions in the temporal lobe. One such MRI finding includes isolated amygdala enlargement, the fourth most common imaging diagnosis in our study, seen in 40 patients (5.4%). Bower et al. [36] found 7/11 MRI negative cases to have unilateral amygdala enlargement retrospectively, concordant with seizure lateralization. Histopathological correlation in one case revealed a glioneuronal hamartoma [36]. Van Paesschen et al. described isolated T2 hyperintensity of the amygdala in 15/31 patients with normal appearing hippocampi, correlating their findings histopathologically to microdysgenesis and gliosis [37].

A total of 35 patients (4.7%) in our cohort presented with tumors including ganglioglioma, DNET, PXA, and LGG. These tumors each have characteristic imaging features; however, overlap has been described. For example, ganglioglioma presents as a cyst with a nodule of contrast enhancement, although it can also appear predominantly solid [38]. DNETs are typically cortically based, intrinsically T2 hyperintense multicystic lesions but can also have variable elements of contrast enhancement [39]. PXAs generally present as cysts with an enhancing nodule plus contrast enhancement of the adjacent meninges [40]. Finally, LGGs are characterized by foci of cortical and subcortical FLAIR hyperintensity that can be located anywhere in the brain and are often difficult to differentiate from FCD [41]. While these tumors are frequently located in the temporal lobe, they are not exclusively temporal entities. In our study, the temporal lobe was involved in 79% of tumor cases. We realize that definitive diagnosis of entities such as tumors is not possible by MRI, and this may have skewed tumor diagnosis or differentiation from FCD in our study.

The temporal lobe was by far the most affected lobe in our study, being either uniquely or partially involved in 58.6% of cases, with MTS being the most common finding as described above. Hippocampal malrotation (or incomplete hippocampal inversion) was found in 9 (1.2%) of our study patients and while appearing to involve the hippocampus itself, through an abnormal triangular or rounded hippocampal shape and vertical collateral sulcus, it is thought to represent a surrogate marker for a developmental lesion elsewhere in the brain. EEG does not necessarily correlate to the side of hippocampal malrotation and the finding can be seen in up to 19% of healthy volunteers [42]. Lesions demonstrating mass effect onto the hippocampus with hippocampal compression were seen in 3 cases in our study cohort (craniopharyngioma, middle cerebral artery aneurysm, and arachnoid cyst). Published case reports describe similar findings, postulating ischemic change in the compressed hippocampus as the nature of the seizure activity [43].

White matter abnormalities in the anterior temporal lobe (WAATL) is another entity gaining interest amongst clinicians in the setting of temporal lobe epilepsy and was found in 8 (1.1%) of our patients. Characterized by increased T2/FLAIR hyperintensity of the temporopolar white matter and loss of gray-white matter differentiation, this entity can present as a dual pathology (with MTS for example) or appear as an isolated finding [44, 45]. In a study with 54 WAATL patients, all 54 lateralized to the same side as the seizure foci in preoperative intracranial EEG and intraoperative electrocorticography [44]. Associated gray and white matter volume loss has also been described in a quantitative study by Coste et al. [46]. Histologically, microdysgenesis and gliosis have been described as a correlate [44]; however, it is debatable whether these changes can account for the increased T2/FLAIR signal. Furthermore, the entity does not appear to demarcate the epileptogenic focus itself as no difference was found in seizure-free outcome whether or not it was resected [47] and it is considered by many to represent a result, rather than a cause, of temporal lobe epilepsy [44, 47].

While encephaloceles are not isolated to the temporal lobe, all of our cases of encephalocele (10) were temporal. Encephaloceles are congenital or acquired in nature, and are usually associated with intracranial hypertension or trauma in adults; spontaneous (idiopathic) encephaloceles have also been described [48]. Operative resection of the encephalocele and the epileptogenic brain tissue can result in total seizure remission [49]. The proposed mechanism of epileptogenicity relates to traction effects onto the herniated brain [50], i.e., pressure inducing ischemia and gliosis. Cases of brain herniating into arachnoid granulations within the transverse sinus have also been described [51]. Encephalocele represents a further entity gaining awareness in previously reported MRI negative cases discovered at surgery [52], with increased detection rates reported through re-evaluation of the MRI upon guidance by seizure semiology [53] and usage of high-resolution T2 weighted sequences (e.g. CISS, SPACE) [54, 55].

Finally, apart from FCD described earlier, a small number of other malformations of cortical development were encountered in our study, including polymicrogyria, periventricular and subcortical nodular heterotopia, band heterotopia, schizencephaly, and hemimegalencephaly. Malformations of cortical development (including FCD) account for up to 40% of pediatric epilepsy cases and while less frequent in the adult population, they still play a significant role in adult MRE [56].

Ultimately, imaging abnormalities must be correlated with a variety of tests including electroencephalography (EEG), positron emission tomography (PET), magnetoencephalography (MEG), video-EEG, neuropsychology testing and other clinical findings. The precise location of seizure origin cannot be determined by MRI alone and can be distant from the lesion identified [57]. In MRI negative surgical candidates, EEG, predominantly in the form of invasive EEG with subdural grid or depth electrodes, can help pinpoint the location of seizure origin and guide resection. While MRI is a pillar in the setting of MRE, it is neither highly sensitive for certain entities nor definitive in the planning of surgical resection and patient care requires a truly interdisciplinary approach at a dedicated tertiary center.

While the lack of histological correlation is a limitation of this study, we felt it necessary to include all patients with MRE to simulate the clinical setting faced by radiologists in routine practice. Excluding patients based on lack of histological correlation would have resulted in exclusion of the greater part of the cohort, and the primary goal of the study was to describe the spectrum of potential imaging findings in this patient population. Second, we did not correlate imaging findings with other modalities such as EEG, MEG or PET, as this often represents a downstream, interdisciplinary step if surgery is being considered for the patient. Thus, the imaging findings reported in this study may not represent the epileptogenic focus on a case-by-case basis, in line with the scenario faced in clinical routine. Furthermore, results from EEG, MEG or PET may not always be available for consultation by the radiologist at the time of reporting.

This study provides an updated description of 3 T MRI findings in adult MRE patients at a tertiary epilepsy center with the goal to familiarize all brain MRI readers with the spectrum and frequency of potential imaging findings in this patient population.

## Data Availability

Data and materials are available upon reasonable request.

## Abbreviations

CISS: Constructive interference in steady state
DNET: Dysembryoplastic neuroepithelial tumor
FCD: Focal cortical dysplasia
LGG: Low grade glioma
MRE: Medically refractory epilepsy
MTS: Mesial temporal sclerosis
PXA: Pleomorphic xanthoastrocytoma
SPACE: Sampling perfection with application optimized contrasts using different flip angle evolution
